# Hemangiopericytome nasosinusien: difficulté diagnostique et thérapeutique

**DOI:** 10.11604/pamj.2014.18.53.3456

**Published:** 2014-05-15

**Authors:** Mohamed Roubal, Aziza Horra, Mohamed Yahya Bajja, Hicham El Ettar, Reda Abada, Sami Rouadi, Mohamed Mahtar, Abdelaziz Janah, Fatmi Kadiri

**Affiliations:** 1 Service d'ORL et de Chirurgie cervico-faciale, Hôpital 20 Août, CHU Ibn Rochd - Casablanca, Maroc; 2 Laboratoire d’étude anatomopathologique moulay Driss Premier- Casablanca, Maroc

**Keywords:** Hémangiopericytome nasosinusien, histologie, Sinonasal Hemangiopericytoma, histology

## Abstract

L'hemangiopericytome est une tumeur vasculaire rare, développée à partir des pericytes des capillaires, dans sa localisation nasosinusienne elle ne représente que 0 .5% de l'ensemble des tumeurs de cette région. Une jeune de 35ans a présenté une tumeur rapidement évolutive au cours du bilan diagnostic, l’étude anatomopathologique a conclu à un hémangiopericytome.

## Introduction

Les hémangiopéricytomes sont des tumeurs vasculaires rares. La région nasosinusienne ne représente que 5% de ses localisations [1]. Ils se développent à partir des péricytes des capillaires. Le premier cas d'hémangiopéricytome nasale a été décrit par Stout et Murray en 1942 [2]. La localisation nasale a des caractéristiques spécifiques qui la différencient des autres hémangiopéricytomes. Elle est moins agressive et a des taux de récidive plus élevés. Ses principales manifestations cliniques sont une épistaxis et une obstruction nasale [3]. Nous rapportons un cas d'un hémangiopéricytome de grande taille de la cavité nasale droite, opéré dans notre service.

## Patient et observation

**Figure 1:**
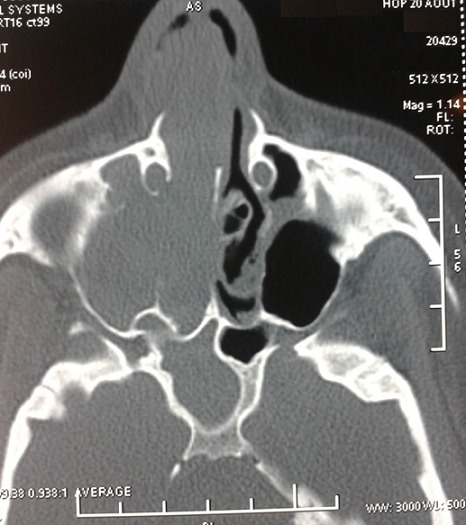
Coupe axiale passant par le sinus maxillaire: Comblement du sinus et de la fosse nasale homolatérale avec lyse de la paroi intersinuso-nasal

Mme T.F., âgée de 35 ans, sans antécédent de traumatisme facial, qui présentait un dysfonctionnement rhinosinusien chronique depuis 6 mois, associant une obstruction nasale et une rhinorrhée antérieure droite, avec des épisodes d’épistaxis. La symptomatologie s'est compliquée 3 mois après par l'extériorisation d'une masse de la fosse nasale droite, qui était partiellement nécrosée et saignante au contact. Un examen tomodensitométrique réalisé avec injection de produit de contraste a mis en évidence un comblement total du sinus maxillaire droit et des cellules ethmoïdales antérieures, laminant et soufflant les parois du sinus maxillaire, s’étendant au niveau de la fosse nasale droite, à travers l'ostium du sinus maxillaire qui est élargi, arrivant au niveau de la choane droite en arrière, au niveau de la narine droite en avant et au niveau des cellules éthmoïdales en haut (Figure 1). La patiente a bénéficié d'une biopsie par voie endonasale sous anesthésie générale, l'acte opératoire était marqué par un saignement important.

**Figure 2:**
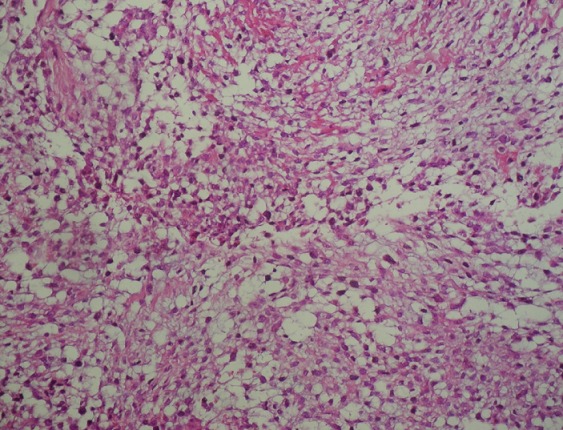
Prolifération de cellules fusiformes

**Figure 3:**
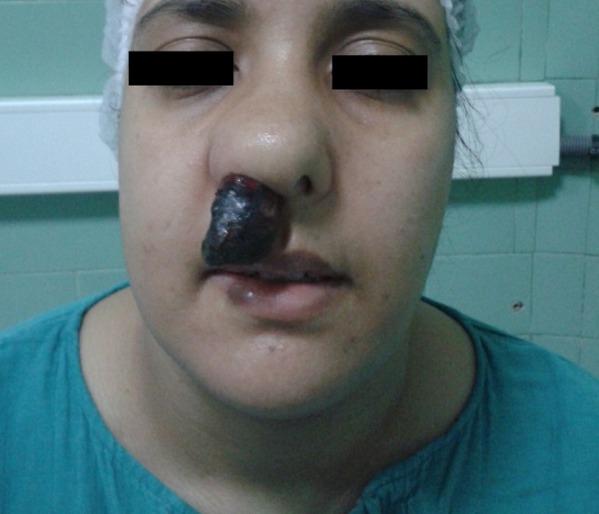
Extériorisation de la masse par la fosse nasale

L'examen anatomo-pathologique avait trouvé des cellules rondes de taille petite à moyenne, à cytoplasme éosinophile large parfois avec des aspects myxoides, se disposant autour de vaisseaux qui sont à paroi musculaire artériolaire épaissie et endothélium turgescent (Figure 2). Une étude immunohistochimique était nécessaire retardant le diagnostic. L’évolution était marquée par une reprise rapide de la croissance tumorale avec son extériorisation par la fosse nasale (Figure 3).

Le résultat de l′étude histologique est revenu positif à la vimentine avec une négativité du CD34, de l'HMB45 et de la PS1000, ainsi était retenu le diagnostic d'un hémangiopericytome sinonasal. La patiente a été opérée par voie vestibulaire supérieure de Rouges Denker où une exérèse de la tumeur a été réalisée avec une maxillectomie partielle droite emportant le tiers droit du palais et les dents correspondantes, avec des recoupes ethmoïdales antérieures, et avec une exérèse d'un prolongement au niveau de la fosse infra temporale. Le saignement a été contrôlé par bistouri électrique bipolaire et par compression par des mèches imbibées par de l'adrénaline diluée. La patiente a été revue en consultation et suivie par des rhinocavoscopies, sans lésion décelable et en rémission totale actuellement avec un recul de 10 mois.

## Discussion

L'hémangiopéricytome nasosinusien est une tumeur d'origine vasculaire, développée à partir de cellules mésenchymateuse à différentiation péricytaire, décrite pour la première fois en 1942 par Stout et Murray [2]. Elle est classée par l'Organisation Mondiale de la Santé (OMS) parmi les tumeurs des tissus mous à potentiel de malignité faible à intermédiaire [4]. C'est une tumeur rare qui représente environ 1% des tumeurs d'origine vasculaire [3]. La localisation au niveau de la tête et du cou représente approximativement 15% des localisations des hémangiopéricytomes [5], et les hémangiopéricytomes nasosinusiens représentent moins de 0,5% des tumeurs des cavités nasales et des sinus para nasaux [4]. Le sexe ratio de ces tumeurs est proche de 1 avec une légère prédominance féminine [4]. Tous les âges peuvent être atteints avec un pic à la septième décennie [4]. Aucun facteur étiologique n'a été mis en évidence, en dehors d'une étude qui a objectivé la présence d'un traumatisme facial dans les antécédents de plusieurs de leurs patients [6]. L'hypothèse physiopathologique évoquée dans cette étude était celle d'une prolifération de vaisseaux capillaires et de péricytes dans les processus de cicatrisation après le traumatisme. Dans le cas de notre patiente il n'y avait pas de notion de traumatisme facial.

Les signes fonctionnels les plus courants sont une obstruction nasale unilatérale et une épistaxis. D'autres signes plus rares ont été rapportés comme des troubles visuels, une otite séromuqueuse, des douleurs de la face et des céphalées [7, 8]. Cliniquement, les hémangiopéricytomes nasosinusiens apparaissent comme des tumeurs polypoïde de couleur rougeâtre à gris rosée. Ce sont des masses molles qui peuvent être ædémateuses ou hémorragiques [4].

Le diagnostic histologique d'hémangiopéricytome est difficile, car cette tumeur n'a pas de profil histologique et immunohistochimique caractéristique. Depuis 2002, l'OMS les classe dans le groupe des tumeurs fibroblastiques et myofibroblastiques [4]. C'est un diagnostic d’élimination pour lequel il faut écarter les diagnostics différentiels qui peuvent partager avec lui des ressemblances histologiques. En effet, s'il n'est pas difficile de distinguer les hémangiopéricytomes des sarcomes qui sont clairement plus malins, la difficulté se pose surtout pour les tumeurs à faible potentiel malin tels que le granulome pyogénique, la tumeur fibreuse solitaire, le léiomyome et l'angiofibrome [9].

Sur le plan immunohistochimique, on peut distinguer les hémangiopéricytomes nasosinusiens de ceux des autres localisations par leur expression accrue de la Vimentine, de l'actine et du facteur XIIIA, et une expression moins importante du CD34. Le Bcl-2, CD99 et CD117 sont négatifs [4]. Dans le cas de notre patiente, l'immunohistochimie montre une positivité de la Vimentine et une négativité du D34 et de la protéine 100.

L'imagerie doit comprendre un examen tomodensitométrique (TDM) pour évaluer l'atteinte osseuse et une imagerie par résonnance magnétique (IRM) pour mieux apprécier les caractéristiques des lésions tissulaires. Ces examens mettent en évidence une lésion tissulaire avec un rehaussement après injection de produit de contraste en TDM, et de gadolinium en IRM [10]. Le rôle de l'angiographie dans la prise en charge des hémangiopéricytomes n'est pas encore bien défini. Cependant beaucoup d'auteurs préconisent une angiographie dans les tumeurs de grande taille, pour une embolisation qui réduirait le risque hémorragique peropératoire [11].

La chirurgie est le traitement de choix des hémangiopéricytomes nasosinusiens. La voie endoscopique est préconisée en première intention en raison de sa plus faible morbidité, à condition de ne pas avoir une extension locorégionale majeure. Dans ce cas la voie d'abord externe est préférée [12].

La place de la radiothérapie et de la chimiothérapie pour les hémangiopéricytomes nasosinusiens reste discutée. La chimiothérapie n'a pas prouvé son intérêt [6]. La radiothérapie externe a parfois été utilisée mais sans études montrant son efficacité [8].

Les hémangiopéricytomes nasosinusiens doivent être considérés comme une entité à part qui diffère des autres localisations par un degré de malignité plus bas, mais avec des récidives fréquentes [12]. Le taux de récidives des hémangiopéricytomes nasosinusiens varie de 7 à 50%, avec un délai moyen de récidive de 6 à 7 ans [11]. Le facteur principal incriminé dans les récidives est le caractère complet ou incomplet de l'exérèse. D'autres facteurs augmenteraient aussi le risque de récidive, tels que la grande taille de la tumeur, l'atteinte osseuse, un pléomorphisme nucléaire sévère et un index mitotique élevé [9, 11]. Dans le cas de notre patiente, on note on atteinte osseuse associée, une taille tumorale supérieure à la moyenne et des marges d'exérèse non saines. Les métastases à distance sont extrêmement rares pour les hémangiopéricytomes nasosinusiens [11], ce qui concorde avec l’évolution bénigne de ces tumeurs avec un taux de survie supérieur à 90% à 5 ans en cas de résection complète de la tumeur [4].

## Conclusion

L'hémangiopéricytome nasosinusien est une tumeur rare d'origine vasculaire, qui diffère des autres localisations d'hémangiopéricytome par son grade de malignité plus bas et son meilleur taux de survie, à condition de réaliser une exérèse chirurgicale complète. La difficulté diagnostique reste un problème majeur d'autant plus que les éléments cliniques ne sont pas spécifiques et l’étude architecturale doit écarter plusieurs diagnostics pour conclure à ce type histologique. Un suivi post opératoire au long court est recommandé pour diagnostiquer les récidives qui peuvent survenir des années plus tard.
